# History of Organophosphorus Compounds in the Context of Their Use as Chemical Warfare Agents

**DOI:** 10.3390/molecules30071615

**Published:** 2025-04-04

**Authors:** Maciej Boczkowski, Stanisław Popiel, Jakub Nawała, Hubert Suska

**Affiliations:** Institute of Chemistry, Military University of Technology, Kaliskiego 2, 00-908 Warsaw, Poland; maciej.boczkowski@wat.edu.pl (M.B.); jakub.nawala@wat.edu.pl (J.N.); hubert.suska@wat.edu.pl (H.S.)

**Keywords:** organophosphorus compounds, chemical warfare agents, Chemical Weapon Convention, OPCW, Novichoks

## Abstract

This is a broad look at the history of phosphorus—from the element through its inorganic and organic compounds to the applications of organophosphates. In addition to commercial and peaceful applications, they were used as chemical warfare agents (CWA), both in military operations and for terrorist purposes. This article attempts to provide a concise history of their development and application in this shameful role. The origin of the chemistry of phosphorus compounds to obtain precursors for the production of CWA is presented. Rapid progress in organophosphorus chemistry in the second half of the 20th century is also described. A broad overview of chemical structures is presented, including lesser-known representatives. The mode of action and the associated toxicity of organophosphorus compounds are briefly mentioned. The Chemical Weapons Convention (CWC) schedules and their changes during their validity are indicated. They are also demonstrated to be used in proficiency tests organised by the Organization for the Prohibition of Chemical Weapons (OPCW). Organophosphates called “Novichok agents”, classified as fourth-generation chemical warfare agents, are also briefly discussed.

## 1. Introduction—General Information About Phosphorus

Phosphorus is a non-metallic element that belongs to group 15 of the periodic table. Its Greek name, phosphorus, means “light-bearer”. The discovery of phosphorus is attributed to Brand, a German alchemist who made it in 1669. Phosphorus comes in several allotropic varieties, the most common being white, red, and black phosphorus. Phosphorus has one stable isotope, P-31. Elemental phosphorus is obtained from phosphates and apatites by reduction with silicon dioxide and carbon in electric furnaces. The raw product is purified by distillation. The raw material obtained in this way is used, among others, for the production of artificial fertilisers and detergents and in the synthesis of precursors for the production of organophosphorus compounds.

The industrial preparation of selected phosphorus derivatives is based on the reaction of phosphorus with chlorine to form phosphorus trichloride and pentachloride—PCl_3_ and PCl_5_, as well as a direct reaction with sulphur, which leads to phosphorus pentasulfide P_4_S_10_. Oxidation of phosphorus trichloride, controlled hydrolysis of phosphorus pentachloride, or its reaction with P_2_O_5_ gives phosphorus oxychloride POCl_3_. Passing gaseous phosphorus trichloride over liquid sulphur gives thiophosphoryl chloride PSCl_3_. It should be emphasised that all industrially important compounds are produced from PCl_3_. These are substances necessary for the synthesis of organophosphorus compounds, particularly to produce CWA [1].

## 2. The Invention of Starting Materials for the Precursor Production to CWA Syntheses of the G and V-Series

The basic precursor for the synthesis of representatives of the G series (The “G” in G-series organophosphorus nerve agents refers to “German”, as these agents were first synthesised in Germany during the 1930s and 1940s) compounds (including sarin and soman, in the CWC assigned to Schedule 1.A.1) is phosphorus trichloride, which is itself included in Schedule 3 of the Convention (States Parties of the Chemical Weapon Convention created a verification regime for certain toxic chemicals and their precursors built in the form of Schedules 1, 2, and 3 in the Annex on Chemicals in order to ensure that such chemicals are only used for purposes not prohibited under the Convention—see: https://www.opcw.org/chemical-weapons-convention, accessed on 26 February 2025). This compound was first described in 1808 by the famous French scientists Gay-Lussac and Thénard. These were the times when scientific discoveries were described in daily newspapers. In the article, they described an experiment that involved the reaction of calomel (Hg_2_Cl_2_) with phosphorus [2]. Phosphorus pentachloride was described by the equally famous scientist Davy at the end of 1808. In his extensive article, he referred to the research of the above-mentioned French scientists [3]. However, Davy incorrectly estimated the composition of this compound. It was not until the French chemist Dulong correctly identified this compound as PCl_5_ in 1816 [4].

The first synthesised compound of the G series is tabun. In the CWC, this class of compounds is included in Schedule 1.A.2. Phosphorus oxychloride was used to synthesise tabun according to the Schrader method. Phosphorus oxychloride was first obtained and described by Wurtz in 1847. The synthesis reaction of this compound was carried out by adding water to phosphorus pentachloride. Interestingly, the chemical notation used by Wurtz was different from the one currently used [5].

Diphosphorus pentasulfide, which can be used to produce compounds of the V series (The “V” in the name of V-series organophosphorus nerve agents refers to “venomous”, reflecting their large potency properties), was first synthesised by the famous Swede Berzelius, in 1843. The reaction of liquid white phosphorus with sulphur at elevated temperature was used [6,7].

**Figure 1:**
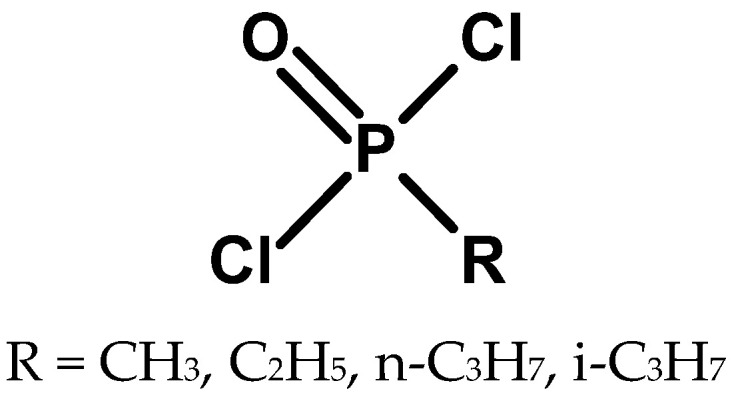
Structure of alkylphosphonic dichlorides that can be used for the synthesis of organophosphorus CWA.

The next step toward the synthesis of organophosphorus CWA was the preparation of alkylphosphonic acid dichlorides (Figure 1). Methylphosphonic acid dichloride was synthesised in 1873 by von Hofman. He obtained it by chlorinating methylphosphonic acid with phosphorus pentachloride [8]. This scientist also obtained ethylphosphonic dichloride [1]. Methylphosphonic, ethylphosphonic, and isopropylphosphonic acids were obtained by oxidation of appropriate phosphines obtained in the reaction of phosphorus trichloride with dimethylzinc with nitric acid. These were among the first compounds with a direct carbon-phosphorus bond [9,10].

## 3. History of Organophosphorus CWA

The French chemist Paul Thénard (son of Louis Jacques Thénard) is credited with the first observation that a compound composed of carbon, phosphorus and hydrogen could exist. In 1845, he described the results of his experiments, where he obtained a mixture of several compounds, including trimethylphosphine [10]. One of the next organophosphorus compounds produced was triethyl phosphate. This was done by the Swiss chemist Franz Voegeli in 1848 [11]. The next synthesised substance of this class was tetraethyl pyrophosphate (TEPP). It was the first obtained in the laboratory organophosphorus compound, which was a cholinesterase inhibitor [11]. The synthesis of TEPP was done in 1854 by Moschnin and de Clermont in the laboratory of Wurtz (who, among other things, researched phosphoric acids). However, the toxic properties of TEPP were discovered only at the turn of the 1930s and 1940s by German scientists, and in 1944, it was introduced for use as a pesticide [1]. It is worth mentioning that Tomasz Miłobędzki worked on methods of synthesising this compound. His achievements include the Polish contribution to research on phosphorus compounds. In addition, in 1897, he investigated the reactions of phosphorus trichloride with alcohols [12].

**Figure 2:**
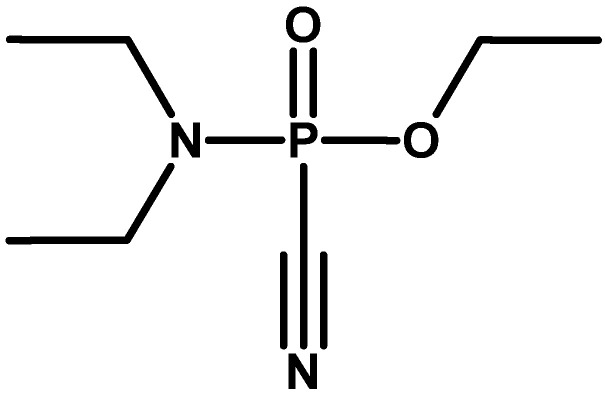
Structure of tabun ethyl analogue.

In 1898, Michaelis, studying the reactions of trialkyl phosphites with alkyl halides, obtained dialkyl phosphonates containing a P–C bond in their structure [13]. Arbuzov examined and described reactions of this type in detail. This process is known as the Arbuzov (Michaelis–Arbuzov) rearrangement. This is one of the basic methods to obtain the P–C bond. A modification of this process is known as the Michaelis–Becker reaction. Also, in 1898, Schall, a doctoral student of Michaelis, obtained ethyl diethylamidocyanide phosphate (Figure 2), an ethyl analogue of tabun, but incorrectly identified its structure [14]. His supervisor correctly defined it in 1903, describing it in a long, 130-page article [15]. Probably due to the low process efficiency or lower toxicity, no toxic effects of this substance were observed.

**Figure 3:**
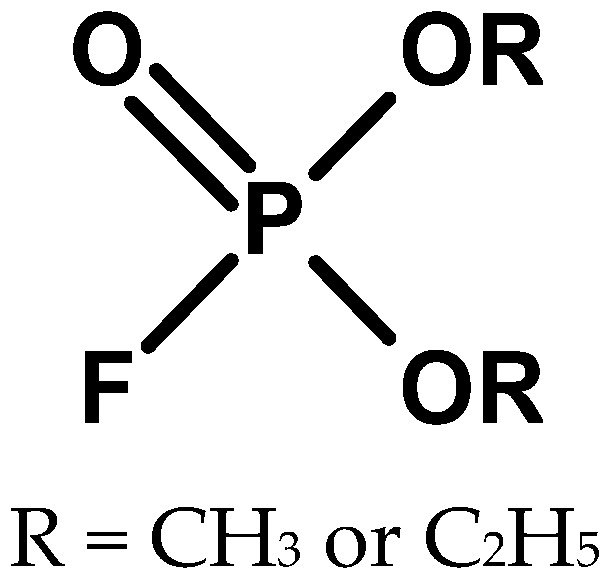
Compounds obtained by Lange and co-workers.

In the 1930s, fluorine became widely available for chemical synthesis, and the German scientist Lange was the first to use it to produce organophosphorus compounds [16]. In 1932, the same scientist, together with Gerda von Krueger, described the toxicity of monofluoroesters of phosphoric acid (O,O-diethyl fluorophosphate and O,O-dimethyl fluorophosphate) (Figure 3) [17].

**Figure 4:**
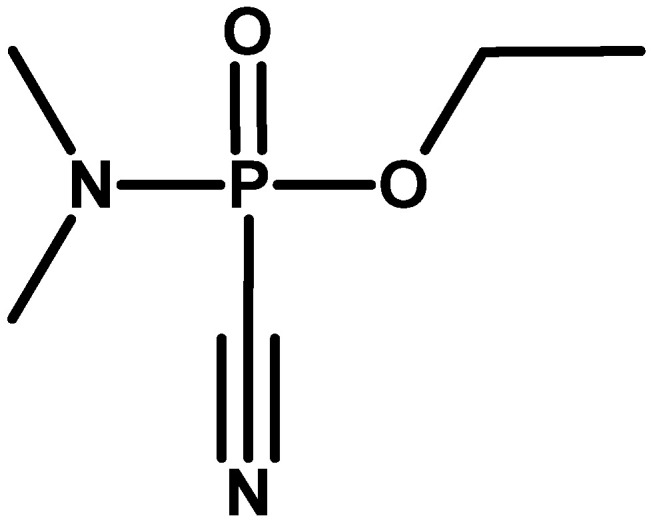
Structure of tabun.

In 1936, Schrader, employed at IG Farben, developed a method for the synthesis of a group of new, highly toxic compounds, derivatives of phosphoric acid, based on the above discoveries [14]. This invention almost cost him his life. One of the compounds, later known as tabun (Figure 4), was so toxic for the conditions of that time that it was classified and considered a potential chemical warfare agent.

**Figure 5:**
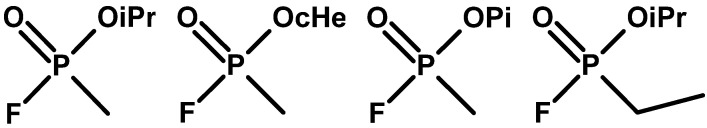
Compounds synthesised during World War II: sarin, cyclosarin, soman, and ethyl sarin. Where -iPr—isopropyl; -cHe—cyclohexyl; -Pi—pinacolyl.

The era of modern CWA has begun. In 1938, another highly toxic compound, called sarin, was synthesised (Figure 5). In 1944, research on toxic compounds was continued at the Kaiser Wilhelm Institute [18] The team was led by Kuhn, the 1938 Nobel Prize winner for his work on vitamins and carotenoids. One of his co-workers was Henkel, later head of the Henkel corporation, who synthesised about ten compounds by esterifying methylphosphonic difluoride [18]. These two scientists are credited with synthesising soman [19]. During World War II, ethyl sarin [20] and cyclosarin [21] were also synthesised (Figure 5).

**Figure 6:**
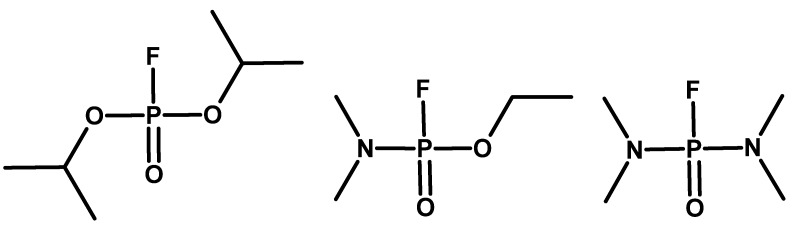
Examples of compounds obtained in the United Kingdom: DFP, fluorotabun, and dimefox.

In the United Kingdom, research conducted by Saunders and co-workers led to the synthesis of DFP (O,O-diisopropyl fluorophosphate) in 1941. In 1942, fluorotabun and dimefox were obtained (Figure 6). The British team was inspired by the works of Lange [18,22,23,24,25,26,27,28,29,30,31].

In the USSR, Kabachnik synthesised sarin in 1944, regardless of German achievements [1]. In the USA, since 1941, extensive research on CWA was carried out in Division 9 (a unit of the National Defence Research Committee, which was an advisory body to the Office of Scientific Research and Development; Division 9 (Chemistry Division) dealt with “chemical warfare problems”), including compounds containing phosphorus. About 200 substances were tested for their suitability as poisonous warfare agents [20] and in 1944, research on the mechanism of enzyme inhibition caused by phosphorus compounds was published [32].

After the war, information about German work on toxic organophosphates captured by the Allied forces was used to develop their own research. In the UK, at the centre in Porton Down, a group of scientists improved the process developed in the USA [33] and later called the Kinnear–Perren reaction (KP reaction)—another way to obtain the P–C bond [34]. Table 1 summarises the most crucial information on progress in phosphorus chemistry. It is a subjective approach of the authors to the issues of phosphorus chemistry. Other essential achievements in this field also exist, but the number of entries in the Table should have been limited.

**Figure 7:**
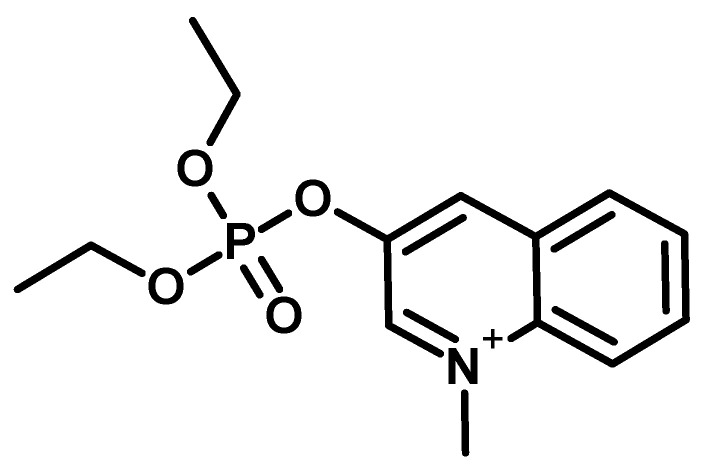
The compound known under the code name of Ro 3-0422.

Research on phosphates was also conducted there (one of the representatives known under the code name Ro 3-0422) (Figure 7), which turned out to have high anticholinesterase activity [35].

**Figure 8:**
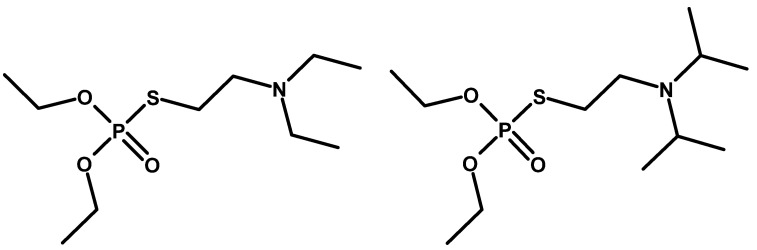
Amiton (**left**) and its isopropyl analogue (**right**).

In the early 1950s, the high toxicity of organophosphorus thiocholine esters was revealed [16]. Experiments carried out in a few centres led to the synthesis of several substances of this type. In 1952, the ICI consortium (International Consortium Initiative) obtained amiton (S-[2-(Diethylamino)ethyl] O,O-diethyl phosphorothioate and codename: VG), used as one of the first organophosphorus pesticides [36], although it is currently considered too toxic for use in agriculture [37]. It can be treated as a precursor of V-series compounds (Figure 8). This is likely why it is listed in the CWC as an exception of Schedule 2.A. Interestingly, its isopropyl analogue (S-[2-(Diethylamino)ethyl] O,O-diisopropyl phosphorothioate) (Figure 8) has higher toxicity and is not included in the Convention lists [38]. Later, other analogues of amiton were also tested [39].

**Figure 9:**
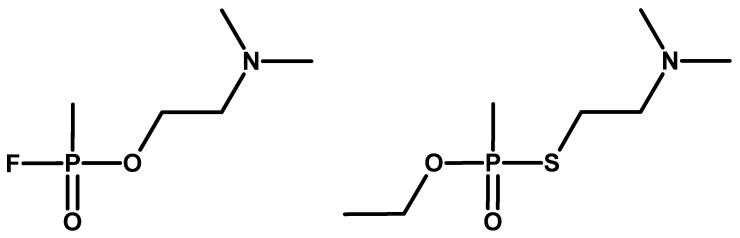
Examples of compounds obtained by Tammelin.

In the 1950s, the Swedish chemist Tammelin developed a method for synthesising a group of compounds later known as Tammelin esters [40], and also synthesised compounds from group V (Figure 9) [41].

In Sweden, research was also carried out on the reactivation of acetylcholinesterase inhibited by these substances [42]. There are also known works by Holmsted, who dealt with the pharmacology of organophosphorus CWA and developed one of the first cross-sectional works on toxic organophosphorus compounds [16]. Research on “Tammelin esters” was also carried out in the then Netherlands [43] and Czechoslovakia [44].

**Figure 10:**
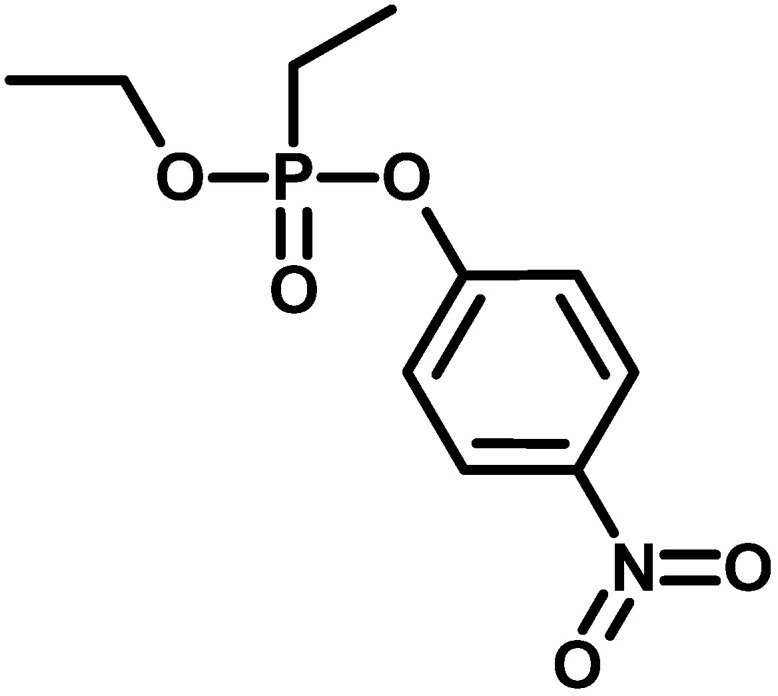
Structure of armine.

**Figure 11:**
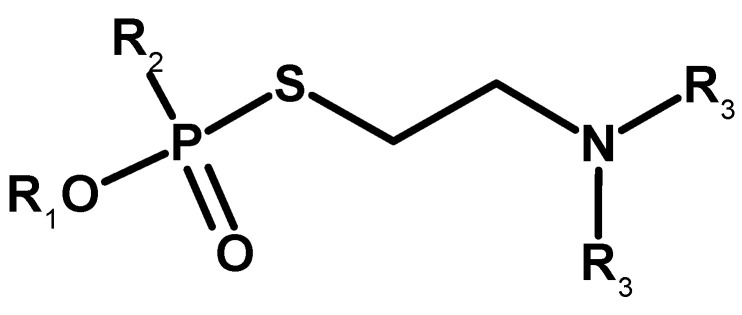
The general structure of the V-series compounds.

Armine, an organophosphorus pesticide described in 1957 (Figure 10), is an ethylphosphonic acid unsymmetrical diester with an ethyl and paranitrophenyl substituent [45]. Little information about this class of compounds is available in the literature, and their mass spectra are not available in commercial libraries. However, they arouse constant interest; for example, a group of researchers from Brazil described a method of synthesising a methylphosphonic derivative, which was then used in research on AChE inhibition as a simulant for VX (IUPAC name: S-{2-[Di(propan-2-yl)amino]ethyl} O-ethyl methylphosphonothioate) (Table 2 and Figure 11) [46].

**Figure 12:**
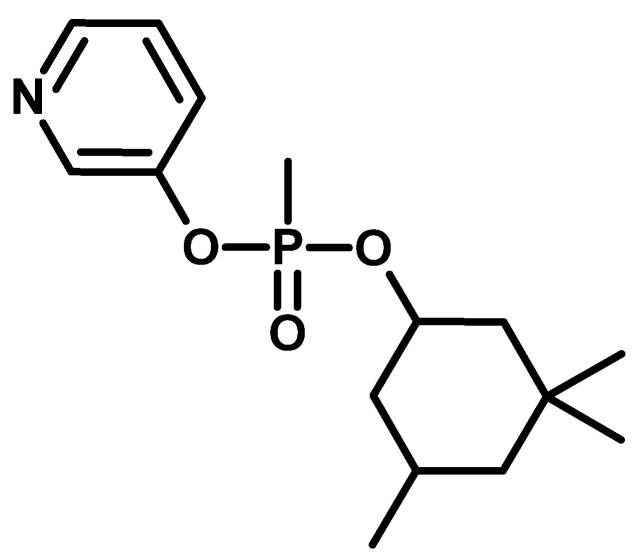
The compound with the code name EA 1511 and the VP symbol.

Research on organophosphorus CWA was carried out on a much larger scale in the USA, among others, at Edgewood Arsenal. A new class of compounds, called the V series, was tested. The 1957 U.S. report by the Army Chemical Corps lists six compounds from group V [47], including one with an unusual structure (Figure 12) [48].

**Figure 13:**
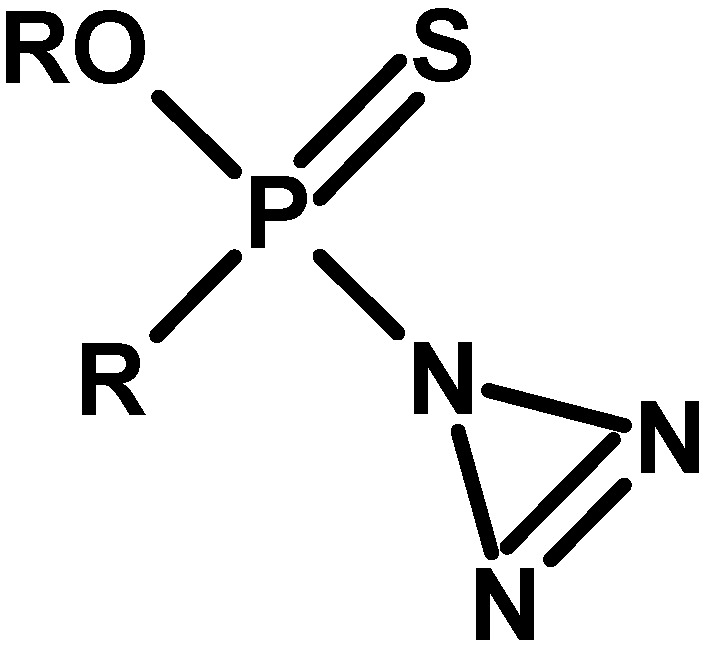
Structure assigned to Subclass IV [50].

The next study, in 1972, provided six generic structures of potentially toxic organophosphorus compounds. In addition to the five structures describing known CWA (including compounds of the G and V series), there is one that is not widely known (Figure 13) [49].

Another report from 1983 listed 47 toxic organophosphorus compounds, including 27 of the G series and 10 of the V series, as well as their precursors and other CWA [50]. These activities resulted in the development and selection of a substance marked with the acronym VX (O-ethyl-S-[2-diisopropylamino]ethyl-methylthiophosphonate) as a new CWA (Figure 11). The work on its binarization was also carried out (as well as the binarization of sarin). Likely based on materials obtained as a result of intelligence activities, similar results were later achieved in the USSR by introducing a compound codenamed R-VX, VXR, or R-33 (S-[2-(Diethylamino)ethyl] O-(2-methylpropyl) methylphosphonothioate) [21]. It is possible that a similar method led to the development of the so-called Chinese VX (C-VX) (O-n-butyl-S-[2-diethylamino]ethyl methylthiophosphonate) [51].

In 1973, a comprehensive study on the problems associated with chemical and biological weapons was published in Sweden [52]. The history of the use and development of toxic warfare agents was presented in detail. It contains much information about organophosphorus compounds, including data on the toxicity of selected substances. However, in this study, which is highly recommended, there is a certain inconsistency: in the first volume (p. 75), the authors did not provide the VX formula, claiming that it is secret, while in the next volume (p. 57) the VX structure was shown [52].

**Figure 14:**
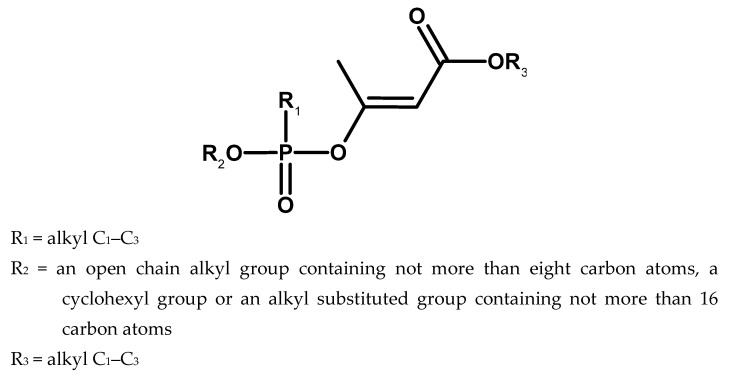
Phosphonate esters of alkyl acetoacetates.

Back in 1969, methods for obtaining phosphonate esters of alkyl acetoacetates were also patented, which were supposed to have high anticholinesterase activity (Figure 14) [53].

Compounds with the following substituents were to be particularly toxic: R_1_ = methyl and R_2_ = cyclohexyl or substituted cyclohexyl. An example is a substance with code EA 1576 (2-ethoxycarbonyl-1-methyl-vinyl cyclohexyl methylphosphonate) [50].

**Figure 15:**
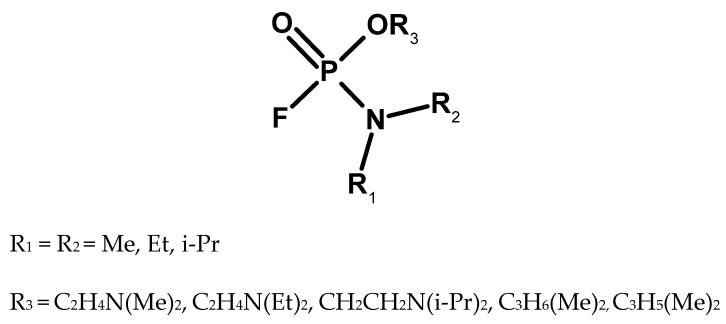
The GV (or IVA) family of compounds [56].

At the end of the 1970s, information about another subgroup of organophosphorus compounds synthesised in the United States appeared. Their representative was N,N-dimethylamido-(N,N-dimethyl-amino-O-ethyl)fluorophosphate, also known as IVA [Intermediate Volatility Agent] and under the code name EA 5365 [50] GV (Organophosphorus GV-series is a group of nerve agents with properties similar to the “G-series” and “V-series”) or GP (Figure 15). This compound was supposed to combine the high volatility of sarin to create high concentrations of the agent over the potential target and the high inhalation and dermal toxicity characteristic of VX [18,54]. It is possible that it was intended to be produced on a larger scale as a binary agent in chemical warheads for MLRS systems [55].

According to other sources, this compound did not have optimal properties due to the introduction of too many toxophore groups into the structure [21]. Research on a group of similar compounds (including IVA) has been conducted since the early 1980s in Czechoslovakia. A number of compounds were synthesised, their structures and basic physicochemical data were provided, and it was noted that they were unstable and suitable for binarization. Studies have also been conducted to determine their toxicity [44,57,58]. Research on this substance was conducted at the beginning of the 21st century in the USA [59].

**Figure 16:**
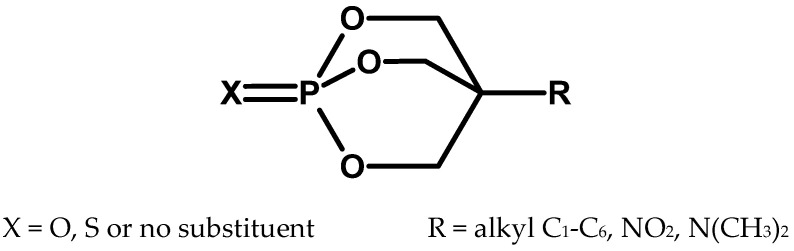
The general structure of bicyclic organophosphorus esters.

In 1973, information about a new group of organophosphorus compounds called bicyclic organophosphorus esters appeared in the Western scientific press (Figure 16). These are solids with a toxicity comparable to that of known CWA. For example, 4-isopropyl bicyclic phosphate (IPTBO) in mice studies turned out to be 33 times more toxic than DFP [60,61,62]. They continue to arouse interest, especially the synthetic routes [63] and the spectral data of two representatives of this group of compounds (4-Isopropyl-2,6,7-trioxa-1-phosphabicyclo[2.2.2]octane1-oxide, and 4-tert-Butyl-2,6,7-trioxa-1-phosphabicyclo[2.2.2] acetate) are included in the National Institute of Standards and Technology (NIST) mass spectral library.

It is worth adding that in the 1970s, it was believed that they did not inhibit acetylcholinesterase but had other toxic effects. Therefore, oxime therapy should have been ineffective and even harmful.

**Figure 17:**
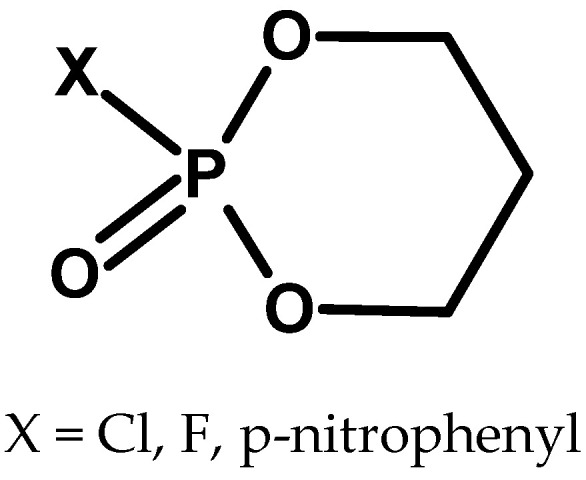
Examples of cyclic halophosphates.

**Figure 18:**
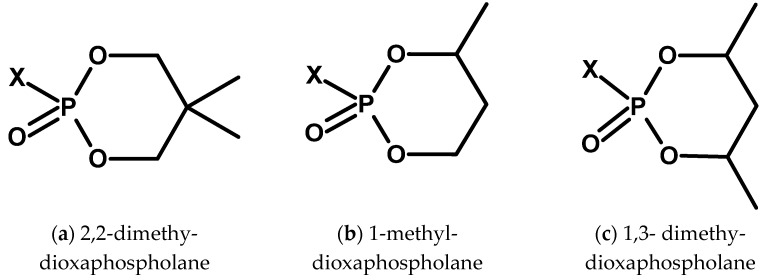
Examples of dioxaphospholane compounds.

At a similar time, articles about cyclic fluorophosphates and chlorophosphates appeared in the context of their biological activity (Figure 17 and Figure 18) [64].

One of the compounds tested was neopentyl fluorophosphate, structurally similar to DFP (Figure 18a) [65].

**Figure 19:**
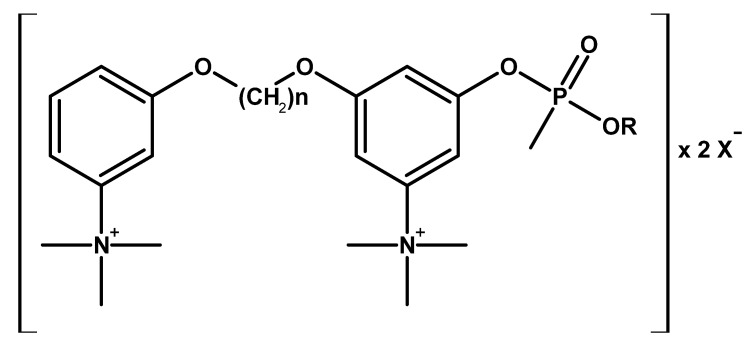
General structure of methylphosphonic acid esters with quaternary ammonium groups.

At the same time, patents were published on compounds being methylphosphonic acid esters with quaternary ammonium groups, which showed high toxicity (Figure 19 and Table 3) [66].

**Figure 20:**
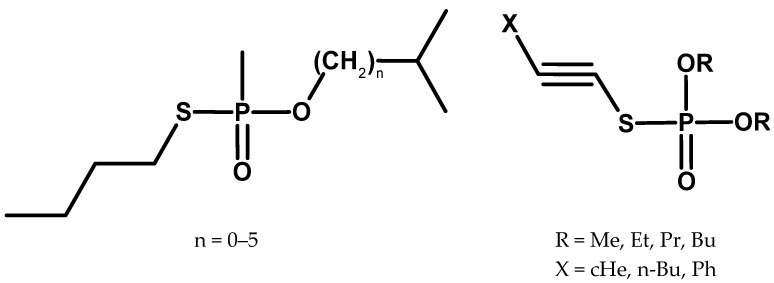
Examples of compounds obtained by Kabachnik’s team.

Since the second half of the 1960s, the Russian scientific press has published articles describing research on broad groups of organophosphorus compounds in the context of their anticholinesterase activity. The research team included, among others, the previously mentioned Kabachnik, Arbuzov’s co-worker [1], known for discovering a process later called the Kabachnik–Fields reaction in the early 1950s, which was used in the chemistry of organophosphorus compounds [67]. He appears to have been an important figure in the Soviet chemical weapons programme. He was one of the founders of the “Kabachnik cholinesterase kolkhoz”, which gathered specialists in the field of research on the broadly understood chemistry and biochemistry of organophosphorus compounds [68]. It is worth adding that in 1946, he was awarded the Stalin Prize of the first class for synthesising sarin and, in 1974, the Lenin Prize for research on V compounds [1,69]. In the research conducted at the “Institute of Hetero-Organic Compounds” of the USSR Academy of Sciences in Moscow, a wide range of substituents were used to modify the basic phosphorus skeleton (Figure 20) [70,71,72,73,74,75,76].

**Figure 21:**
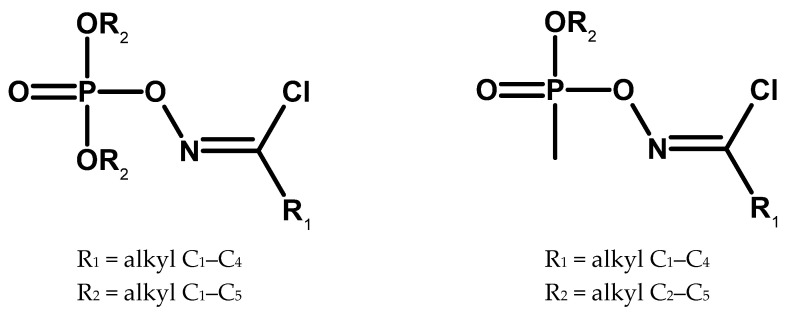
Examples of compounds obtained by the team of the Institute of Physiologically Active Compounds.

Another research team at the “Institute of Physiologically Active Compounds” of the USSR Academy of Sciences in Moscow conducted similar research. A member of this team was Martynov, one of the winners of the Lenin Prize in 1972 for the synthesis of soman [69]. However, the focus was on slightly different compounds, phosphorylated oximes, described since the mid-1960s (Figure 21) [77,78,79,80,81,82].

The Russian scientific press continuously publishes articles describing research on a wide range of organophosphorus compounds, also in the context of their biological activity [83].

**Figure 22:**
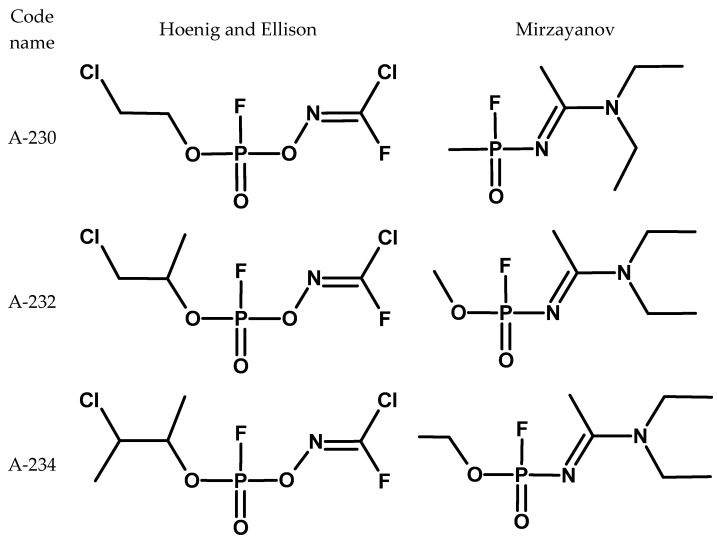
Comparison of the basic structures of “Novichoks”, proposed by Hoenig and Mirzayanov.

According to some sources, since the 1960s, work was carried in the USSR out under the programme codenamed “Foliant” on compounds later called “Novichok agents”, or compounds A and classified as fourth-generation CWA [84]. However, information on this subject is incomplete and unverified. These substances, described as phosphorylated oximes, were supposed to be highly toxic, resistant to antidotes, and undetectable by standard detectors [21,85]. In addition, common organophosphorus precursors used in the production of fertilisers or pesticides could be used for their syntheses [86]. There was considerable controversy about their structure. Western authors provided one [56,87] (Hoenig was not sure of the proposed structures), and their Russian whistleblower, V. Mirzayanov, provided others (Figure 22) [84]. Historically, both groups of compounds were called Novichoks. However, it was the OPCW that clarified and defined which group of compounds are Novichoks and included them in the CWC. These compounds were described by Mirzayanov.

**Figure 23:**
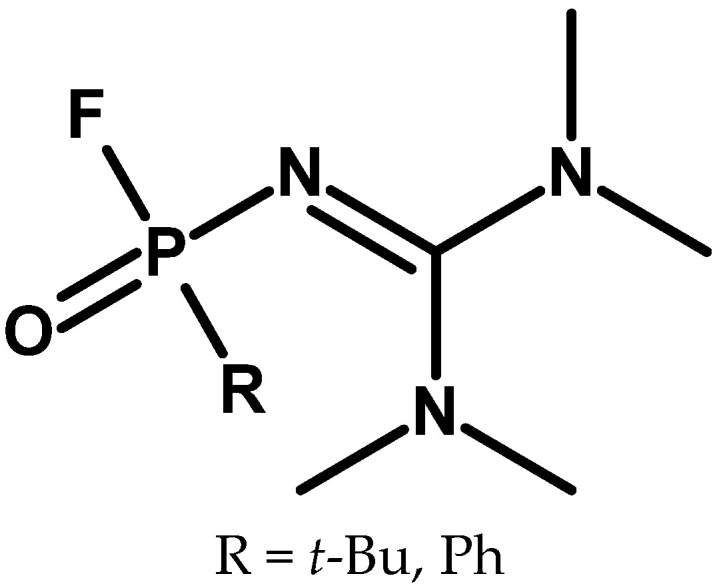
Compounds described by Münchenberg.

The first article that described the synthesis of compounds of this class appeared only in 2016 [88]. However, a work on synthesising substances with a similar structure was published in 1996 [89]. It should be noted that one of the substances described there, in the case of where a methyl substituent is used as R, will be a tetramethyl derivative of the compound currently known under the code name A-242 (Figure 23).

Subsequently, review articles were published [90,91,92,93]. Research publications have also been published [94,95,96,97,98]. Research on the simulation of mass spectra, physical properties, and toxicity has also been conducted [99,100,101]. The international political consequences of using these substances to assault human life resulted in updating Schedule 1 of the CWC in 2019 [102]. This happened for the first time since its inception in 1993. Mass spectra of several representatives of this class of compounds were placed in 2019 in library provided by the OPCW Technical Secretariat: OCAD v.21 [103].

**Figure 24:**
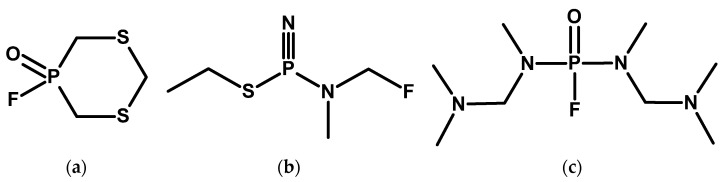
Examples of compounds described in Ledgard’s manuals: (**a**) Actinkinlum (NPF-512), (**b**) Xansten, and (**c**) Lisvonfox (LL-59). The structures shown represent compounds from outside the CWC.

In 2003, the first edition of Ledgard’s “manual” [104] was published, which is cited as a source of recipes for the synthesis of direct precursors for the production of CWA [88]. The second was published in 2007 [105], the third in 2012 [106], and the fourth edition in 2022 [104]. The last two editions contain descriptions of the syntheses of many substances that have not been described so far in the generally available literature (Figure 24). Procedures for obtaining their precursors and detailed diagrams of the equipment used are also provided. Selected data (e.g., molecular weight, density) are also included. The analysis of the structures of these compounds and the given properties suggest that they are highly toxic.

There are papers that describe further studies on compounds already known. However, mass spectra are poorly documented in libraries [107,108]. Importantly, information about research on new classes of compounds continues to appear [109,110], and the basic structure is being modified by introducing atoms of other elements that are not often used in the synthesis, such as selenium or tellurium [89]. Although the toxicity of selenium derivatives was already described in the 1960s [111], publications with extensive research on this subject have only recently appeared [112,113]. Table 4 describes the most important events in the field of CWA syntheses.

## 4. Briefly on the Toxicity of Organophosphorus Compounds

The toxic properties of organophosphorus compounds were discovered in the 1930s in Germany. Their toxicity was so powerful that they were secretly proposed and developed as chemical weapons [51]. The use of organophosphorus compounds as insecticides began in the 1940s and received a significant boost between 1960 and 1980 when they gradually replaced organochloride insecticides (which are less toxic but much more persistent in the environment) [114]. It should be clearly emphasised that their high environmental instability and sensitivity to hydrolytic degradation allow them to be used relatively safely in agriculture. Over 100 types of organophosphorus pesticides and herbicides are currently used in agriculture in different parts of the world, particularly in developing countries. The specific organophosphorus pesticides used vary between regions, depending on the climate, cost, country regulations and controls [115].

Organophosphorus nerve agents are widely recognised as chemical poisons and are among the most deadly chemical poisons ever developed (Table 5 and Table 6). The main target of organophosphorus toxins (OPT), acetylcholinesterase (AChE), hydrolyses acetylcholine (ACh) and is mainly associated with nerves and muscles, being typically found on the synapses [51]. OPT, including nerve agents, produce acute toxicity primarily by inhibiting AChE, disrupting the homeostatic regulation of acetylcholine-dependent cholinergic signalling in the central and peripheral nervous system.

The failure to metabolise or break down ACh causes an accumulation of ACh at the nerve terminals, leading to increased cholinergic stimulation and many of the toxidromic symptoms typical of nerve agent poisoning, known medically as a cholinergic crisis. Increased cholinergic stimulation leads to persistent stimulation of muscarinic receptors within the parasympathetic neurons. In mammalian species, parasympathetic nerves innervate the airways. Muscarinic receptors belong to the G-protein coupled receptor family presenting throughout the airways. Overstimulation of muscarinic receptors initiates increased salivation, lacrimation, urination, and defecation (commonly known as SLUD), along with bronchoconstriction, bradycardia, increased nasal secretions, emesis, and dyspnea. Nicotinic receptors, a ligand-gated ion channel family member, occur in the somatic or sympathetic nervous system and are also affected by OPs. Symptoms include tachycardia, mydriasis, fasciculations, meiosis, skeletal muscle paralysis, hypertension, slurred speech, irritability, fatigue, impaired judgment, insomnia, and diaphragmatic weakness. More severe poisoning is associated with more profound central nervous system responses such as ataxia, convulsions, seizures, and death by asphyxiation [118].

All nerve agents present optical isomers: tabun, sarin, cyclosarin, VX, and VR have one chiral phosphorus atom each, while soman has an additional stereocenter at a carbon atom of the pinacolyl group. These stereoisomers react with AChE at different rates and possess distinct toxicological properties [119]. These differences result from the stereochemistry of the active site of AChE, which favours the accommodation of one enantiomer at the active site to the detriment of finding another one.

One of the significant differences between the nerve agents is associated with the AChE enzyme. When a nerve agent inhibits acetylcholinesterase (AChE), it initially forms a stable covalent bond between the phosphorus and serine oxygen atoms in the enzyme’s active site. Over time, a secondary reaction called ageing occurs, involving the cleavage of one of the side groups, usually an alkoxy group, from the nerve agent molecule attached to AChE. This process stabilises the phosphorylated enzyme, making it irreversible and unresponsive to reactivation by common oxime antidotes such as pralidoxime (2-PAM). The order of t_1/2_ for the ageing of human AChE nerve agent adducts was found to be, in ascending order, soman (2 min), sarin (3 h), cyclosarin (7 h), tabun (19 h), and VX (36.5 h). Due to the rapid ageing caused by agents like soman, prompt diagnosis and treatment are essential. For slower-aging agents such as VX, oximes can remain effective much longer. The ageing of inhibited AChE is described as another enzyme-catalyzed reaction despite the enzyme being covalently modified by a nerve agent [120].

Treatment for OP intoxication includes atropine, a muscarinic receptor antagonist, an anticonvulsant such as diazepam, and acetylcholinesterase reactivator, an oxime. Some AChE reactivators, such as bispyridinum oximes, HI 6 and HLö 7 with atropine, are quite effective. Oximes can reactivate acetylcholinesterase by attaching to phosphorus, subsequently divorcing from the AChE enzyme. Many other oximes were also investigated [121].

Although AChE inhibition can be simplistically discussed as an irreversible reaction, detailed kinetic studies account for spontaneous reactivation, releasing the active enzyme and hydrolysed detoxifying agent [122].

In addition to acute toxicity, many OP compounds produce delayed neuropathy in humans, which develops 8–14 days after poisoning. Weakness and ataxia develop in the lower limbs and can progress to paralysis, which, in severe cases, can also affect the upper limbs. The severity of the neuropathy depends upon the compound and the dose absorbed. Recovery is slow and is seldom complete. Poisoning results in the distal degeneration of some long axons in peripheral nerves and spinal cord [123]. Table 5 and Table 6 present selected organophosphorus CWA toxicity data.

## 5. Use of Organophosphorus CWAs

Organophosphorus CWA has been used in wartime and peacetime as terrorist agents or for executions or murders. The first fully documented use of organophosphorus CWA occurred in 1984 during the Iraq–Iran war. The Iraqis used then tabun (and other CWA) [124]. At this time, CWAs were also used in the form of a mixture in 1988 during the infamous attack on the city of Halabja.

On 27 June 1994, in Japan (Matsumoto incident), the Aum Shinrikyo sect carried out an attack using sarin gas on judges participating in a trial against members of the sect. Seven people died (the eighth remained in a coma for 14 years and died in 2008), and over 200 were reversibly injured. On 20 March 1995, perhaps the most famous case of chemical terrorism occurred. A group of members of the Aum Shinrikyo sect mentioned here carried out a sarin gas attack on five subway trains in Tokyo. Approximately 5.5 thousand people were poisoned, 12 of whom died. It should be emphasised that the attack occurred during the morning rush hour and was calculated so that all contaminated trains met at the Kasumigaseki junction station almost simultaneously.

**Figure 25:**
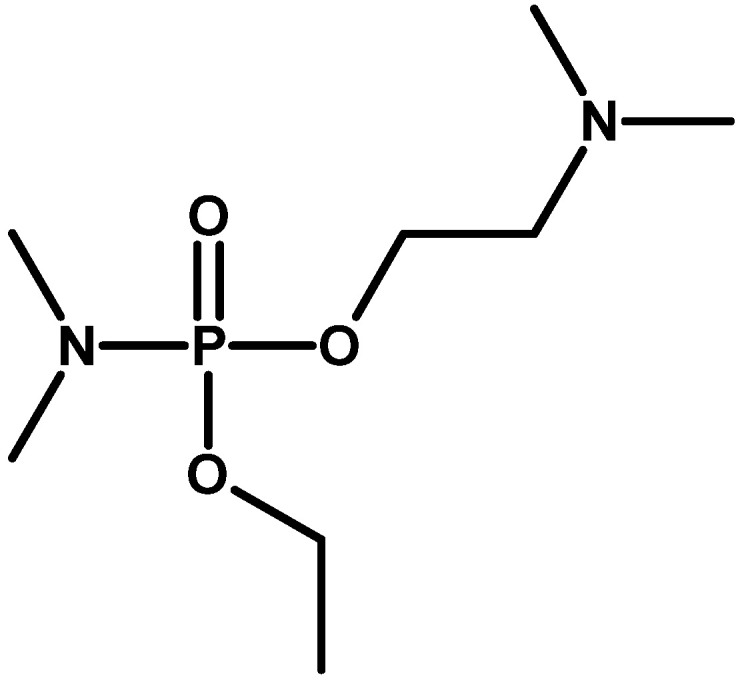
The compound described in the two reports.

It is suspected that an organophosphorus compound (with a structure similar to the compounds of the GV series) was used in another terrorist attack in August 1995 [125,126,127]. The structure of this compound shown in Figure 25 represents a chemical outside of the CWC.

In August 2013, sarin was used during the Syrian civil war, causing many civilian deaths. The UN report confirms this [128]. Syria was forced to ratify the CWC in September 2013 following this event. Renewed attacks using this agent occurred in April 2017, causing additional victims [129].

## 6. Chemical Weapons Convention

### 6.1. Historical View

The use of poisons in combat is as old as human history. There have been many attempts to limit or exclude this method of fighting. The first major legal act in this field was the Fourth Hague Convention of 1907 [130]. However, the experiences of World War I showed the weakness of its solutions. It was then developed in the Geneva Protocol of 1925 [131]. Two world wars and the arms race that emerged after World War II (including chemical weapons) are proof that this document also does not live up to the hopes placed in it.

International negotiations on the implementation of the Chemical Weapons Treaty began at the UN Conference on Disarmament in 1980. The talks took place mainly in Geneva. International negotiations on the implementation of the Chemical Weapons Treaty began during the UN Conference on Disarmament in 1980. The talks took place mainly in Geneva. It can be debated why this work was initiated: whether it was to altruistically rid the world of the threat posed by highly toxic substances or raise awareness on the part of the powers possessing chemical weapons that retaliation might be taken by other powers who also possessed chemical weapons at the time, as happened in the First World War. Working meetings were also held in the United States (1986, Tooele) and the USSR (1987, Shikhany). On 30 November 1992, in New York, the UN General Assembly adopted the Chemical Weapons Convention by Resolution A/RES/47/39 [132], the full name of which is as follows: Convention on the Prohibition of the Development, Production, Stockpiling and Use Chemical Weapons and the Destruction of Their Stockpiles. The adopted text of the Convention was presented for signature in January 1993 in Paris [133]. The date of entry into force of the Convention was not determined until 31 October 1996, when Hungary became the 65th state to ratify. As required, the Convention entered into force 180 days later, on 29 April 1997. The abbreviated name is the Chemical Weapon Convention—(CWC) [134]. The Organisation for the Prohibition of Chemical Weapons (OPCW) was established as the executive body of the Convention. The introduction of this act of international law was undoubtedly a big step towards freeing the world from the dangers caused by one of the representatives of weapons of mass destruction.

### 6.2. Content of the Convention Lists (In Terms of Organophosphorus Compounds)

An important component of the Convention is its Schedules. They were organised into three groups, each with two subgroups; the first is toxic substances (marked with symbol A), and the second is precursors (marked with symbol B). Schedule 1.A is particularly important because it groups the highly toxic substances. It can be considered a list of compounds used only for military purposes, although the Convention does not define Schedule 1.A is a closed list of CWAs. Until June 2020, Schedule 1, Part A, included three groups of compounds containing phosphorus, corresponding to the analogues of sarin (1.A.1), tabun (1.A.2), and VX (1.A.3). Part 1.B show the precursors for the production of organophosphorus CWA: four phosphonic difluorides (1.B.9), phosphonites—precursors for the synthesis of compounds V-series (1.B.10), as well as chlorosarin (1.B.11) and chlorosoman (1.B.12). Schedule 1 also includes poisonous agents with a blistering effect (sulfur mustards, nitrogen mustards, and lewisites), as well as saxitoxin and ricin polypeptide [135].

Schedule 2.A includes amiton (2.A.1), a compound initially planned as a pesticide but withdrawn from use due to its high toxicity. Part 2.B includes, among others, precursors for the synthesising of compounds from Schedule 1. List 2.B.4 is particularly extensive because its definition implies that it is practically unlimited. Schedule 2 also includes phosphoramide dihalides (2.B.5) and the corresponding diesters (2.B.6). These are precursors for the syntheses of compounds from list 1.A.2. The subsequent lists include compounds that can be used as precursors for the production of type V compounds (2.B.10, 11, 12) [136].

Schedule 3.B contains the basic precursors for producing organophosphorus CWA: phosphoryl trichloride, phosphorus trichloride, phosphorus pentachloride, and di- and tri-methyl and ethyl esters of phosphorous acid. Additionally, Schedule 3.A contains compounds used as chemical weapons during World War I: phosgene, cyanogen chloride, hydrogen cyanide, and chloropicrin [137].

### 6.3. Changes to the Convention in 2020

It seems that from the beginning of the Convention, there was a need to supplement its lists. After Mirzayanov published his memoirs in 2008 [84], this need was increasingly considered, also in the OPCW. Within the Scientific Advisory Board (SAB), an advisory body of the OPCW, already in 2011, there were discussions about “Novichoks”, as well as GV group compounds and carbamates [138]. There was a definite need to update the lists of the Convention after the sensational events in Great Britain (the case of the poisoning of Skripal and his daughter, 4 March 2018, “the Salisbury Incident”). Consequently, the United Kingdom requested technical support from the OPCW for the above events. The result was information about the identification of a toxic substance [139]. In May 2018, the Director General asked the SAB for information on new types of nerve agents [140]. The answer was given in early July and contained interesting information [141]. In June, another accident involving the use of suspicious substances occurred (30 June 2018, “Amesbury Incident”). Also, on this occasion, the United Kingdom asked for technical support. The use of a toxic substance, the same as during the events in March, was again found [142]. In October this year, the USA, Canada and the Netherlands jointly proposed supplementing List 1 with two groups of compounds (the first with the P–C bond, the second with the P–O–C bond) [143].

Russia’s counterproposal appeared in early December, was more extensive, proposed five groups of compounds, cited scientific literature and patents on carbamates, and provided compounds with CAS numbers. There was also mention of a new class of compounds with anticholinesterase activity, described in the literature as “di(polyfluoroalkyl) fluorophosphates and fluorophosphonates” [144]; however, after the final findings were not introduced into Schedule 1 of the Convention [145]. The consultation process and evaluation of the proposals began in December 2018 [146].

On 27 November 2019, by decision of the Conference of States Parties, changes to Schedule 1 were established [147,148] and in December, a compact text of the new changes was presented [102]. The Russian proposal was accepted, and carbamates were also included. The changes are effective from 7 June 2020, and the Schedule 1 has been updated with four new items:

1.A.13—P-alkyl (H or ≤C10, incl. cycloalkyl) N-(1-(dialkyl(≤C10, incl. cycloalkyl)amino))alkylidene (H or ≤C10, incl. cycloalkyl) phosphonamidic fluorides and corresponding alkylated or protonated salts,
1.A.14—O-alkyl (H or ≤C10, incl. cycloalkyl) N-(1-(dialkyl(≤C10, incl. cycloalkyl)amino))alkylidene (H or ≤C10, incl. cycloalkyl) phosphoramidofluoridates and corresponding alkylated or protonated salts,
1.A.15—Methyl-(bis(diethylamino)methylene)phosphonamidofluoridate,
1.A.16—Carbamates (quaternaries and bisquaternaries of dimethylcarbamoyloxypyridines).

Despite the extension of the content of Schedule 1, some groups of highly toxic compounds containing phosphorus are still outside of it. Some are outside the Schedules of the Convention altogether, for example, GV-type compounds (information on this subject was recorded already in 2002) [59], certain phosphates (including DFP and bicyclic compounds), polyfluoroalkylfluorophosphates and polyfluoroalkylfluorophosphonates [149,150,151]. In the context of the “Navalny case”, information appeared about the use of a substance with a structure similar to the structure of compounds from lists 1.A.14 and 1.A.15, acting as a cholinesterase inhibitor, and being outside the lists of the Convention [152].

### 6.4. How Many Chemicals Are on the Schedules of the Convention?

At the beginning of the Convention’s validity, it was realised that a broad spectrum of chemical compounds would be covered by it. Estimates from 2004 showed that the theoretical amount of organophosphorus CWA in Schedule 1 was approximately 270,000. People were also aware of the extraordinary length of list 2.B.4 (Table 7) [153].

**Figure 26:**
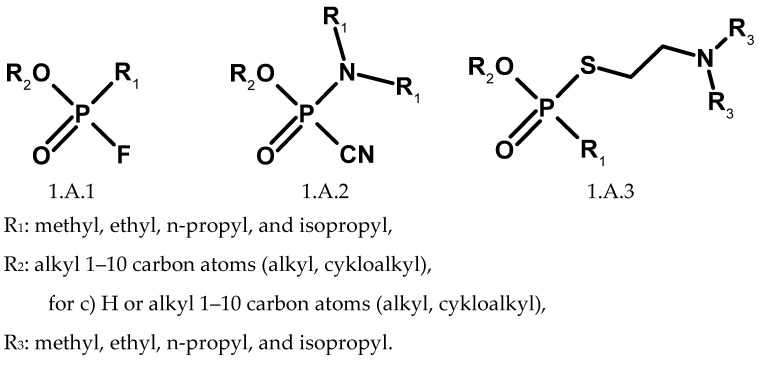
Generic structures of organophosphorus compounds from Schedule 1.A.

**Figure 27:**
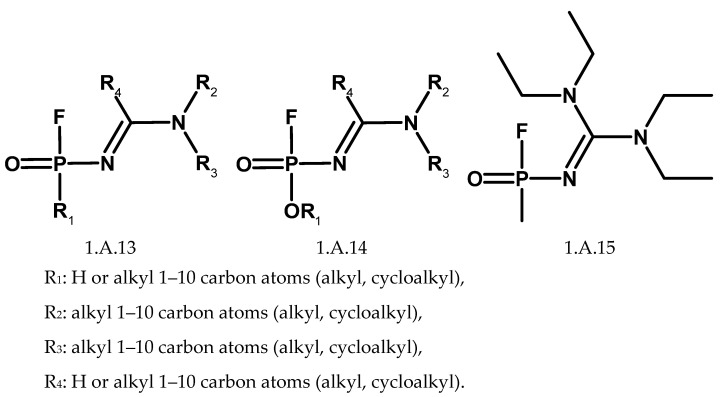
Generic structures of organophosphorus compounds introduced to Schedule 1.A in 2020.

Currently, higher estimates of the number of compounds in the Convention Schedules are provided [154,155,156,157]. Organophosphorus compounds included in lists 1.A.1, 1.A.2, and 1.A.3 are defined by structures that allow for any combination of substituents defined as alkyl or cycloalkyl R_1_, R_2_, and R_3_ (Figure 26). The P-alkyl and N-alkyl substituents are limited to methyl, ethyl, n-propyl, and isopropyl. Alkyl groups bonded to the nitrogen atom may be symmetrical or asymmetrical. Counting the possible variants of the O-alkyl substituent R_2_ (1–10 carbon atoms) gives 580,799 possible structures, although many are unstable. Taking into account all possible combinations (including theoretical ones), list 1.A.1 contains more than 2 million compounds, and list 1.A.2 has more than 5 million. List 1.A.3 includes protonated and quaternary alkyl salts of compound V, but the number of carbon atoms in the alkyl substituent is not specified. This generates an indefinite number of possible compounds. Similar considerations also apply to precursors from list 1.B.10. Analogous problems concern compounds from the newly introduced lists 1.A.13 and 1.A.14 (Figure 27). C1 to C10 acyclic alkyls alone yield 86,760 theoretically possible dialkylamino groups when all symmetric and asymmetric structures are taken into account. Again, the list includes protonated and quaternary alkyl salts. Another issue is the existence of optical isomers and isotopically labelled compounds, which are not distinguished for the purposes of the convention [158].

Similar problems apply to undefined quaternary Amiton derivatives and generic precursor structures for preparing organophosphorus compounds included in lists 2.B.4, 2.B.10. 2.B.11, and 2.B.12. The particularly extensive Schedule 2.B.4 includes all compounds (except those included in Schedule 1) whose molecules contain a phosphorus atom that is directly bonded to one methyl, ethyl, n-propyl, or isopropyl group. This creates a huge, almost infinite number of compounds, especially since only one substituent is defined, and phosphorus can have an oxidation state of +3 and +5. Schedule 3 creates a closed and clearly defined group of compounds.

Even though only a few substances from these lists were produced to be used as chemical weapons, the other compounds remain within the scope of interest of the Convention [153].

### 6.5. Organophosphates in Proficiency Tests Organised by the OPCW

**Figure 28:**
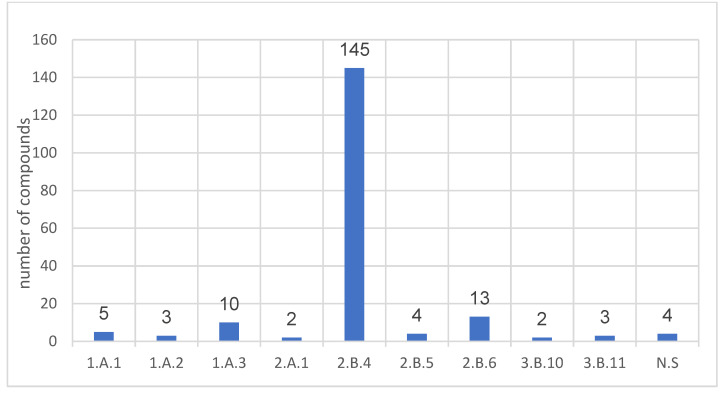
Distribution of organophosphorus compounds used in Proficiency Tests 1–55 as “spiking chemicals”.

The establishment of the OPCW resulted in the need to create a method to verify the provisions of the Convention. This was resolved by initiating and developing a global system of designated laboratories. This has been carried out since 1996 by conducting Proficiency Tests (PT). The test involves analysing the samples provided and preparing a report. Conventional and non-conventional compounds that may originate from the transformation of conventional compounds are used as analytes. The PTs are intended to simulate environmental samples. Matrices are usually organic liquids or water; solids are also sometimes used. After 55 tests, it turns out that approximately half (191 out of 376) of the “spiking chemicals” (substances deliberately added to the samples) are organophosphates. By dividing the analytes into their assigned lists, the following summary was obtained (Figure 28).

## 7. Conclusions

There is no doubt that the history of organophosphorus CWA is long and intriguing. Scientific works without the synthesis of new toxic compounds are constantly being published on testing both known substances and new groups of potentially toxic compounds. These papers mainly describe the decontamination process or spectral and retention data to identify both known substances and their potentially toxic analogues. What is more significant is that we are constantly witnessing incidents where these substances are used to assassinate human lives. In light of continuously published research and the observed effects of the use of these agents, a picture of organophosphorus CWA chemistry emerges as a still-open branch of science.

Despite widespread adherence to the Chemical Weapons Convention, chemical agents still pose a threat due to their potential use by states that do not respect international law or by terrorist groups. Among the many types of CWA, the neurotoxic organophosphates are the most dangerous. To date, no universal antidote for these compounds is known, and the possibility of ageing requires a fast-acting AChE reactivator (especially in the case of soman). Given the above information, it is important to be aware that organophosphorus CWAs are still a significant threat to human life and the environment.

## Figures and Tables

**Table 1:** Selected events in the history of phosphorous and organophosphorus compounds.

Year	Event	Inventor
1696	Discovery of phosphorus—as white phosphorus	Alchemist H. Brand [1]
1808	Synthesis of phosphorus trichloride	J. L. Gay-Lussac, L. J. Thénard [2]
1808	Synthesis of phosphorus pentachloride	H. Davy [3]
1843	Synthesis of diphosphorus pentasulfide	J. Berzelius [6]
1847	Synthesis of phosphorus oxychloride	A. Wurtz [5]
1873	Synthesis of methylphosphonic dichloride	A. W. von Hofman [8]
1898	Michaelis’ reaction	A. Michaelis [13]
1904–1906	Arbuzov’s research	A. Arbuzow [1]
1932	Synthesis of compounds with a P-F bond	W. Lange [17]
1951–1952	Kinnear–Perren reaction	J. Clay, A. M. Kinnear, E. A. Perren [33,34]
1952	Perkow reaction	W. Perkow [1]
1952	Kabachnik–Fields reaction	M. Kabachnik, E.K. Fields (independently) [1]
1967–1969	Allen–Millar–Trippett rearrangement	D. W. Allen, I. T. Millar, S. Trippet [1]

**Table 2:** Representatives of the V-series compounds illustrated by the general formula in Figure 11.

	VX	R-VX	C-VX	VE	VM	VS	VG	EA-3148	EA-2192
R_1_	Et	iso-Bu	n-Bu	Et	Et	Et	Et	cyclo-Pe	H
R_2_	Me	Me	Me	Et	Me	Et	O-Et	Me	Me
R_3_	iso-Pr	Et	Et	Et	Et	iso-Pr	Et	Et	iso-Pr

**Table 3:** Representatives of compounds illustrated by the general formula in Figure 19.

Code Name	R	n	X
EA 2012	iso-C_3_H_7_	4	2 B(C_6_H_5_)_4_^−^
EA 2054	iso-C_3_H_7_	3	2 B(C_6_H_5_)_4_^−^
EA 2098	C_2_H_5_	3	2 B(C_6_H_5_)_4_^−^
EA 2613	C_6_H_13_ (pinacolyl)	3	2 I^−^

**Table 4:** Chronology of the syntheses of toxic organophosphorus compounds.

Year of the First Synthesis	Chemical	Inventor(s)	Source
1854	Tetraethyl pyrophosphate	W.P. Moschnin	[11]
1898	Ethyl analogue of tabun	A. Schall	[14]
1932	Organophosphorus diesters	W. Lange, G. von Kruger	[17]
1936	Tabun	G. Schrader	[14]
1938	Sarin	G. Schrader w/co-workers	[16]
1941	DFP (and derivatives)	B. Saunders w/co-workers	[27]
1944	Soman	R. Kuhn, K. Henkel	[18]
1952	Amiton	R. Ghosh, J. F. Newman	[36]
1952–1957	VX	R. Ghosh	[51]
1969	Phosphonate esters of alkyl acetoacetates	U.S. patent owners	[53]
1960–1990	Binary VX (BIGEYE bomb)	U.S. Armed Forces	[56]
1975	Highly toxic phosphates	U.S. patent owners	[66]
1970–1980	GV (IVA)	U.S. Armed Forces (?)	[18]
1976	Binary sarin (M687 155 mm sarin shell)	U.S. Armed Forces	[56]
1970–(?)	Novichok agents	Former USSR (?)	[84]

**Table 5:** Acute inhalation lethality for nerve agent vapour [116].

Agent	Species	LC_50_ (mg/m^3^)	Duration (h)
GB	Rat (f)	18.1	0.16
GB	Rat (f)	6.39	1
GF	Rat (f)	25.2	0.16
GF	Rat (f)	5.49	1
VX	Rat (f)	5.44	0.16
VX	Rat (f)	0.74	1

**Table 6:** Toxicological parameters of G-, V-, and “A”-agents [117] (The data given in that table was taken from Nepovimova and Kuca review article where they did not provide any information on the species and route of administration and were transferred into their article as such).

Agent	LCt_50_ (mg × min/m^3^)	LD_50_ Percutaneous Administration (mg/Person)
Sarin	100	1700
Soman	70	350
VX	50	10
A-232	6–10	1–2
A-234	7	5

**Table 7:** Estimated number of possible compounds that can be derived from the definitions for CWC compounds included in Schedules 1, 2, and 3 [153].

Schedule 1	Estimated Number of Compounds	Schedule 2	Estimated Number of Compounds	Schedule 3	Number of Compounds
1.A.1	˃20,000 ^a)^	2.A.1	1	3.A.1	1
1.A.2	˃50,000 ^a)^	2.A.2	1	3.A.2	1
1.A.3	˃200,000 ^a)^	2.A.3	1	3.A.3	1
1.A.4	9	2.B.4	millions	3.A.4	1
1.A.5	3	2.B.5	20 ^b)^	3.B.5	1
1.A.6	3	2.B.6	100	3.B.6	1
1.A.7	1	2.B.7	1	3.B.7	1
1.A.8	1	2.B.8	1	3.B.8	1
1.A.13	??? ^c)^	2.B.9	1	3.B.9	1
1.A.14	??? ^c)^	2.B.10	10	3.B.10	1
1.A.15	1	2.B.11	8	3.B.11	1
1.A.16	??? ^c)^	2.B.12	10	3.B.12	1
1.B.9	4	2.B.13	1	3.B.13	1
1.B.10	˃200,000 ^a)^	2.B.14	1	3.B.14	1
1.B.11	1	-	-	3.B.15	1
1.B.12	1	-	-	3.B.16	1
-	-	-	-	3.B.17	1

^a)^ Including branched chains and cycloalkanes, excluding bicycloalkanes and stereoisomers, and excluding protonated and alkylated forms. ^b)^ Only dichlorides and difluorides (bromides and iodides also possible) [153]. ^c)^ Supplementing the table with new lists (according to the Russian side’s requests, there may be approximately 300–400 compounds from lists 1.A.13 and 1.A.14, and approximately 400 from 1.A.16) [144].

## Data Availability

Data are made available by the authors upon request.
